# Genetic Diversity Evaluation of Shanghai Local Pig Breeds Using Liquid-Phase Chip Technology

**DOI:** 10.3390/ani16030479

**Published:** 2026-02-03

**Authors:** Mengqian Cao, Jun Gao, Shushan Zhang, Weilong Tu, Lingwei Sun, Jiehuan Xu, Mengqian He, Jianjun Dai, Caifeng Wu, Defu Zhang

**Affiliations:** 1 Institute of Animal Science and Veterinary Medicine, Shanghai Academy of Agricultural Sciences, Shanghai 201106, China; 13887223118@163.com (M.C.); gaojun@saas.sh.cn (J.G.); zhangshushan@saas.sh.cn (S.Z.); tuweilong@saas.sh.cn (W.T.); sunlingwei@saas.sh.cn (L.S.); jiehuanxu@saas.sh.cn (J.X.); hemengqian@saas.sh.cn (M.H.); daijianjun@saas.sh.cn (J.D.); 2 Shanghai Municipal Key Laboratory of Agri-Genetics and Breeding, Shanghai 201106, China; 3 Key Laboratory of Livestock and Poultry Resources Evaluation and Utilization, Ministry of Agriculture and Rural Affairs, Shanghai 201106, China; 4 Shanghai Engineering Research Center of Breeding Pig, Shanghai 201106, China

**Keywords:** SNP liquid-phase chip, genetic diversity, genomic breed composition

## Abstract

This study developed a 60K single-nucleotide polymorphism (SNP) liquid-phase chip (‘Shenxin I’) based on genotyping by target sequencing (GBTS) technology. The chip was used to genotype 1451 individuals spanning five conserved local pig breeds: Fengjing (FJ), Meishan (MMS), Pudong White (PD), Shaowutou (SW), and Shanghai White (SHW), and newborn purebred Meishan offspring (MMS_New). The study assessed the genetic diversity, population structure, and genomic breed composition (GBC) of these breeds. The GBC analysis provided insights into genomic introgression within admixed individuals. Results indicate that PD and FJ breeds exhibited the higher fraction of the genome covered by runs of homozygosity (F_ROH_) and slower linkage disequilibrium (LD) decay rate, suggesting higher genetic inbreeding level in these two breeds. The study conducted genetic distance of identity-by-state (IBS) analysis and molecular pedigree construction for the MMS population. Subsequently, purebred mating was implemented by selecting boars from different pedigrees to mate with sows and excluding individuals with high inbreeding coefficients from the breeding program. This resulted in a significant reduction in the overall inbreeding level of the born progeny and an increase in genetic diversity compared to the original population. Consequently, the study concludes that utilizing liquid-phase chip technology for genotyping, constructing molecular pedigrees, and optimizing mating combinations in small populations of locally conserved pig breeds contributes to enhancing the conservation effectiveness.

## Simple Summary

This study employed genotyping by target sequencing (GBTS) genotyping technology to conduct genetic evaluations of conservation populations for five local pig breeds in the Shanghai region of China, providing theoretical guidance for optimizing breeding strategies within these populations.

## 1. Introduction

As one of the earliest domesticated animals, pigs provided a stable meat supply for human populations and contributed to the development of agriculture [1,2]. China, an early center of pig domestication, possesses rich porcine germplasm resources. According to the National Catalogue of Livestock and Poultry Genetic Resources (2021 edition), China is home to 83 indigenous and 25 cultivated pig breeds, which together constitute approximately one-third of the world’s pig genetic resources.

However, the expansion of livestock genetic resource exchange and the promotion of intensive farming models have led to a sharp decline in the population size of China’s indigenous pig breeds, with some now facing imminent extinction [3,4]. Shanghai is home to a rich variety of indigenous pig breeds. These include four renowned Taihu lake pig breeds—Meishan, Fengjing, Shaowutou, and Pudong White—valued for their high fertility, superior meat quality, and strong adaptability [5], as well as the cultivated Shanghai White pig, another nationally important breed [6]. Prolonged closed breeding within shrinking core populations has heightened several risks, including the erosion of genetic diversity, increased inbreeding, and the decline in key performance traits. Therefore, strengthening the conservation of Shanghai’s indigenous porcine genetic resources and conducting comprehensive studies on their genetic diversity are essential for safeguarding biodiversity and ensuring the sustainable development of the local livestock industry.

Studying the population genetic diversity of indigenous pig breeds provides insights into their origins, evolution, and genetic relationships among different breeds, which is crucial for their conservation and utilization [7]. Conventional livestock genetic evaluation in livestock, largely dependent on phenotypic assessment and pedigree analysis, is often constrained by long cycles, high costs, and limited efficiency. These limitations can cause genetic evaluations to lag behind the dynamic needs of modern breeding programs [8]. The rapid evolution of molecular biological technologies have driven a paradigm shift, with high-throughput genotyping platforms leveraging single-nucleotide polymorphism (SNPs) emerging as transformative tools for genetic evaluation [9,10]. Traditional solid-phase chips rely on probe-DNA hybridization and are analyzed via fluorescently labeled probes, resulting in high costs and limited adaptability for personalized applications. In contrast, genotyping by target sequencing (GBTS) liquid-phase chips offer a cost-effective, scalable, high-throughput platform with exceptional marker density. This technology holds substantial potential to revolutionize genetic breeding models in animal agriculture [11,12].

This study employed the porcine genotyping liquid-phase chip (‘Shenxin I’), developed by our team based on the genotyping by target sequencing (GBTS) [13] technology, to conduct comprehensive genetic diversity analyses of five representative pig breeds in Shanghai, China. The primary objective of this study is to analyze and compare the genetic diversity of Shanghai local pig breeds, conduct a comprehensive individual GBC examination, and provide effective measures for selection in conservation breeding at Shanghai local pig conservation farms. The findings provided theoretical support for the conservation, breeding, and utilization of Shanghai pig genetic resources.

## 2. Materials and Methods

### 2.1. Sample Collection

A total of 1451 samples from conservation populations were collected from the breeding farm of state-level or municipal-level designated conservation units. The cohort comprised 364 Fengjing pigs (FJ), 333 Middle size Meishan pigs (MMS), 397 Pudong White pigs (PD), 104 ShaWutou pigs (SW), 199 Shanghai White pigs (SHW) and 54 MMS newborn piglets (MMS_NEW) (Table 1). The samples consisted of EDTA anticoagulated blood or ear tissues.

### 2.2. The Design of Porcine 60K SNP Liquid-Phase Chip

The porcine 60K SNPs, designated ‘Shenxin I’, developed in this study comprises two modules: an 18.9K SNP panel for genomic breed composition (GBC) analysis and a 41.9K SNP set of breeding-relevant loci derived from commercial pig breeds, as previously established by molbreeding Biotechnology Co., Ltd. (Shijiazhuang, Hebei Province, China) (https://www.molbreeding.com/, accessed on 1 August 2025). This combined design ensures the chip’s broad applicability across diverse pig breeds. The development of the GBC module involved whole-genome resequencing data from 150 representative individuals across different pedigrees from five local pig conservation farms in Shanghai [6] combined with integrated genotype data from over 1200 individuals representing 25 distinct pig breeds (Table 2). This pooled dataset enables GBC evaluation for Shanghai local pig breeds. An initial screening of the original VCF file was conducted to exclude low-quality variant sites, applying the following criteria: a site omission rate below 0.1 and a heterozygosity rate below 0.5. For quality-controlled SNP sites, the following metrics were calculated for each breed: △MAF = |MAF_target − MAF_other|, where MAF_target: the reference minimum allele frequency for the SNP site in the target variety; MAF_other denotes the combined reference minimum allele frequency for SNP sites across all other breeds after excluding the target breed. SNP sites were ranked in descending order of △MAF values. Final selection considered both high △MAF values and uniform chromosomal distribution and coverage.

### 2.3. Validation of the Liquid-Phase Chip

The accuracy of GBC predictions was assessed using three approaches. First, an admixture model based on maximum likelihood estimation using the ADMIXTURE v1.3.0 software [14]. The ancestral cluster parameter K was set to 25 (representing the model was set to 25 breeds). Second, two machine-learning algorithms (Random Forest [15,16] and Multilayer Perceptron [17]) were employed to predict the GBC of the samples. In both cases, 70% of the samples were randomly selected as the training set, while the remaining 30% served as the test dataset. Finally, the predictions from all three methods were compared against the known breed identities to evaluate their agreement.

### 2.4. GBTS Pipeline for SNP Typing

Genomic DNA was extracted from samples, fragmented, and used to construct sequencing libraries through end-repair, A-tailing, adapter ligation, and PCR amplification. The libraries were then subjected to a targeted enrichment via probe hybridization, followed by a post-capture PCR. Library concentration and quality were assessed using Qubit and qPCR before sequencing. The subsequent data analysis pipeline included quality control, alignment to a reference genome, and variant calling/annotation. This entire Genotyping by Targeted Sequencing (GBTS) workflow was performed by MolBreeding Biotechnology Co., Ltd.

### 2.5. Genetic Analysis

#### 2.5.1. SNP Filtering

Quality control was performed using PLINK v1.90 [18] with the following SNP filtering parameters: (1) removal of individuals with a call rate < 90%, (2) selection of autosomal loci only, (3) retention of SNP markers with a detection rate ≥ 90%, (4) retention of SNPs with a minimum allele frequency ≥ 0.01, and (5) exclusion of markers with a Hardy–Weinberg equilibrium test *p*-value of less than 10^−6^ [19].

The minor allele frequency (MAF) [20], proportion of polymorphic markers (PN) [18], runs of homozygosity (ROH) [21], linkage disequilibrium [22] (LD) calculations along with their corresponding r^2^ values, expected heterozygosity (He), and observed heterozygosity (Ho) [23] were calculated in PLINK v 1.90. The following parameters were used when defining ROH: (1) the minimum length of ROH is 500 kb; (2) each sliding window contains at least 30 SNP loci; and (3) a maximum of 5 deletions and 1 heterozygosity are allowed in ROH. The formula of inbreeding coefficient F_ROH_ is $\sum L_{\mathrm{ROH}}$/L_AUTO_, in which L_ROH_ is the total length of the autosomal ROH intervals, and L_AUTO_ is the total length of the autosomes.

Additionally, the polymorphic information content (PIC) [24] and nucleotide diversity (Pi) [25] were calculated using R. The results were visualized and collectively analyzed to assess the genetic diversity across the five Shanghai pig breeds. Raincloud plots for the F_ROH_ statistical indicators in the five pig populations were obtained using OmicShare tools, an online platform for data analyses (http://www.omicshare.com/tools) (accessed on 15 December 2025) [26]. Principal component analysis (PCA), a standard method for inferring population structure and admixture, was performed using PLINK v 1.90. The results were visualized with the ggplot2 package (v. 3.4.0) in R (v. 4.2.2).

#### 2.5.2. Purebred Breeding Selection Between Molecular Pedigrees

The study conducted an IBS analysis and molecular pedigree construction for the MMS population. It classified it into seven molecular families after employing boars and sows from non-related families for pure breeding and excluded a few individuals with relatively high F_ROH_ > 0.3. The purebred offspring covered all boar families, totaling 14 litters. From each litter, 1–2 boars and 2 sows exhibiting sound physical condition were selected for germplasm conservation, totaling 54 individuals (MMS_NEW). Genotyping was performed using the ‘Shenxin I’. This study aims to compare whether genotyping based on molecular pedigrees or pure line selection enhances genetic diversity in subsequent generations.

## 3. Results

### 3.1. Porcine 60K SNP Liquid-Phase Chip

**Figure 1:**
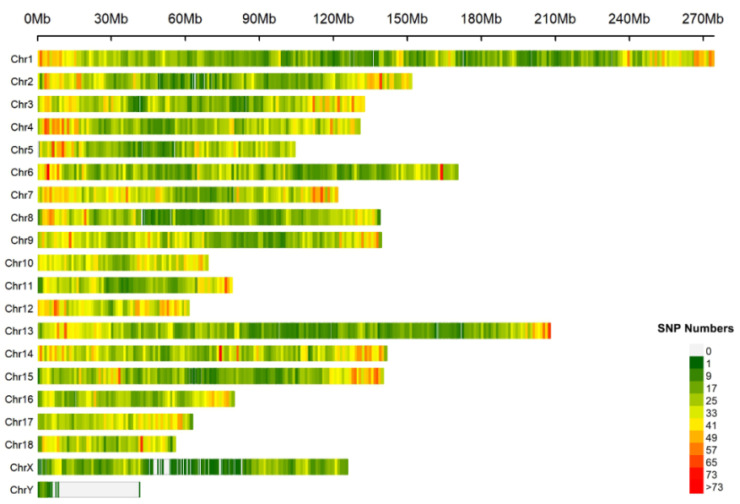
Chromosome distribution of “Shenxin I” SNPs on pig reference genome.

**Figure 2:**
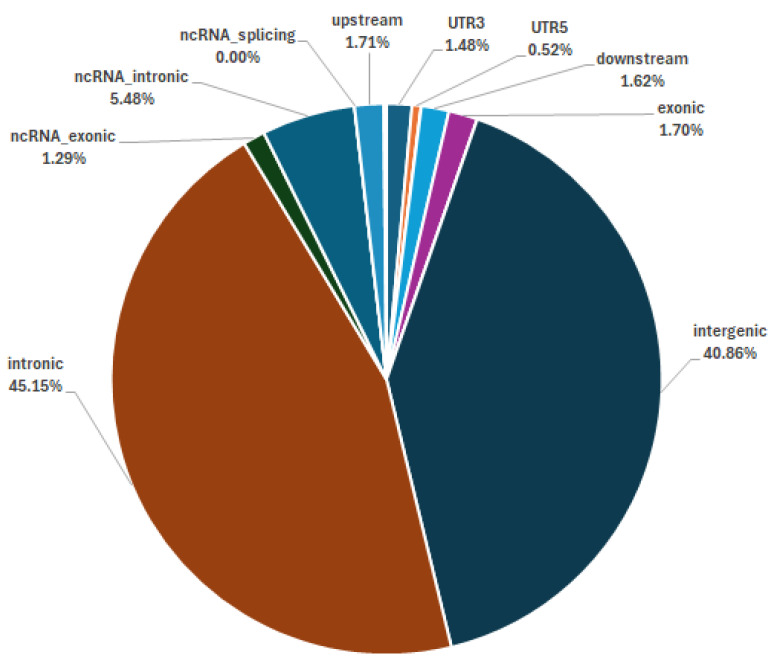
Annotation statistics of the 60K SNPs.

This study designed and produced the porcine ‘Shenxin I’ 60K SNP liquid-phase chip for genetic analysis. The GBC analysis module, approximately 1200 SNPs were selected per chromosome. Following remove deduplication, this yielded an 18,945 (18.9K) SNPs module. By filtering population samples, a standard reference population dataset comprising 25 breeds and 1248 individuals with 18.9K SNP genotypes was ultimately established. This dataset serves as the basis for subsequent GBC analysis of unknown test samples. All 60K sites are evenly distributed across the chromosome (Figure 1) and identified on pig reference genome (*Sus scrofa* 11.1). ANNOVAR v20200607 annotation of the 60K SNP sites detected on the chip revealed that the sites were distributed as follows: 40.86% in intergenic regions, 1.70% in exonic regions, 45.15% in intronic regions, and 1.71% in upstream promoter regions (Figure 2).

### 3.2. GBC Analysis for Conservation Populations

Using software PLINK v1.90 and Admixture v1.3.0, the study extracted genotypes for 18.9K GBC module from the genotyping results of five populations. These were integrated into the established standard reference population genotype dataset (25 pig breeds 1248 individuals) for analysis.

**Figure 3:**
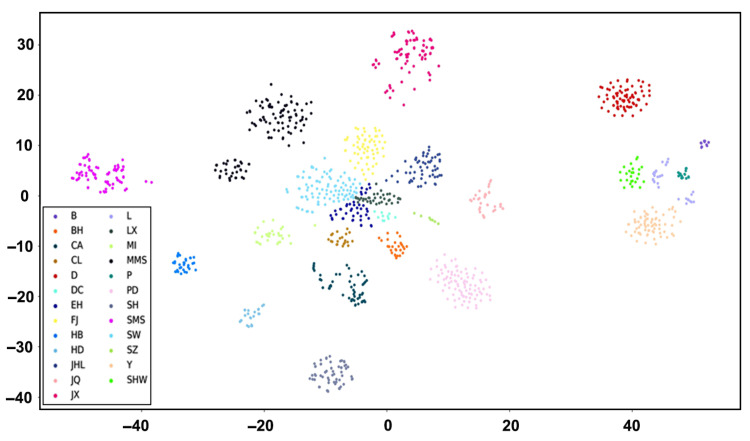
Differentiation of 18.9K SNPs for 1248 samples based on tSNE analysis.

To evaluate the breed-discriminatory power of the selected 18.9K loci, t-distributed stochastic neighbor embedding (tSNE) [27] was applied to the genotype data of all 1248 reference individuals. The resulting low-dimensional projection demonstrated clear separation among breeds, confirming the strong discriminatory capability of the SNP panel (Figure 3). For genomic breed composition (GBC) prediction, the maximum likelihood estimation-based admixture model, along with machine-learning models based on Random Forest and multilayer perceptron, achieved GBC prediction accuracy of 99.6%, outperforming both the Random Forest (99.2%) and multilayer perceptron (98.13%) models. Collectively, these results validate the 18.9K SNP module developed in this study as a highly accurate and suitable tool for GBC analysis of Shanghai local pig breeds.

GBC analysis results based on admixture method of five local pig breeds indicated that the SW population exhibited GBC purity exceeding 99%. Within the FJ population, only one individual scored below 95%, while five individuals were identified in the PD population, seven in the MMS population, and fifty-eight in the SHW population, respectively.

### 3.3. Genetic Diversity of Populations

Genetic evaluation of the five local pig breeds from Shanghai showed that the minor allele frequency (MAF) of SNP markers across populations ranged from 0.2233 to 0.2438. The Shanghai White (SHW) population exhibited the highest mean MAF (0.2438 ± 0.1479), followed by the Pudong White (PD), Meishan (MMS), Shaowutou (SW), and Fengjing (FJ) populations. With the exception of the FJ population, the observed heterozygosity (Ho) in the other four breeds exceeded the expected heterozygosity (He). The FJ population displayed the lowest values for both Ho (0.2954 ± 0.1559) and He (0.2966 ± 0.1543), whereas the PD population showed the highest (Ho: 0.3385 ± 0.1529; He: 0.3295 ± 0.1460).

**Figure 4:**
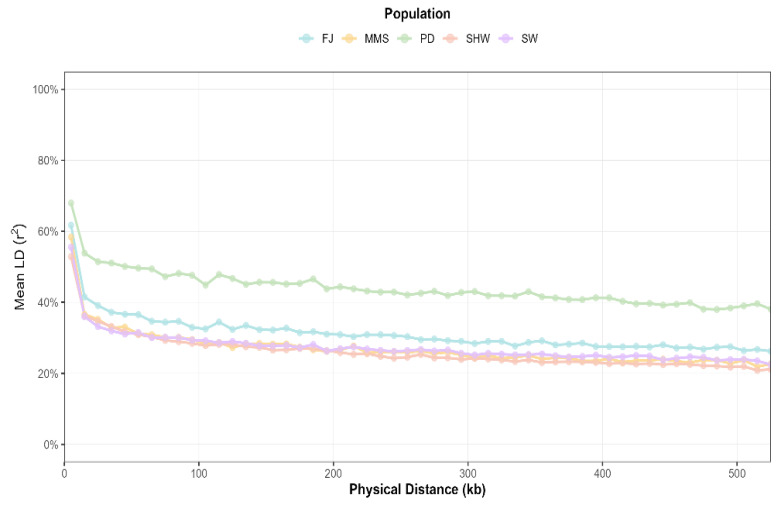
Linkage disequilibrium (LD) decay plots. The horizontal axis represents the physical distance in kilobases, and the vertical axis represents the average degree of LD (r^2^).

Nucleotide diversity (Pi) was highest in the PD population (0.3326 ± 0.1344) and lowest in the FJ population (0.3051 ± 0.1468). Based on autosomal genome data, the polymorphic marker ratio (Pn) was lowest in the PD population (0.4876) and highest in the SHW population (0.7844) (Table 3). Polymorphic information content (PIC) was also calculated for all five breeds. Furthermore, the PD and FJ populations exhibited a slower linkage disequilibrium (LD) decay compared to the other breeds (Figure 4).

### 3.4. Principal Component Analysis (PCA)

**Figure 5:**
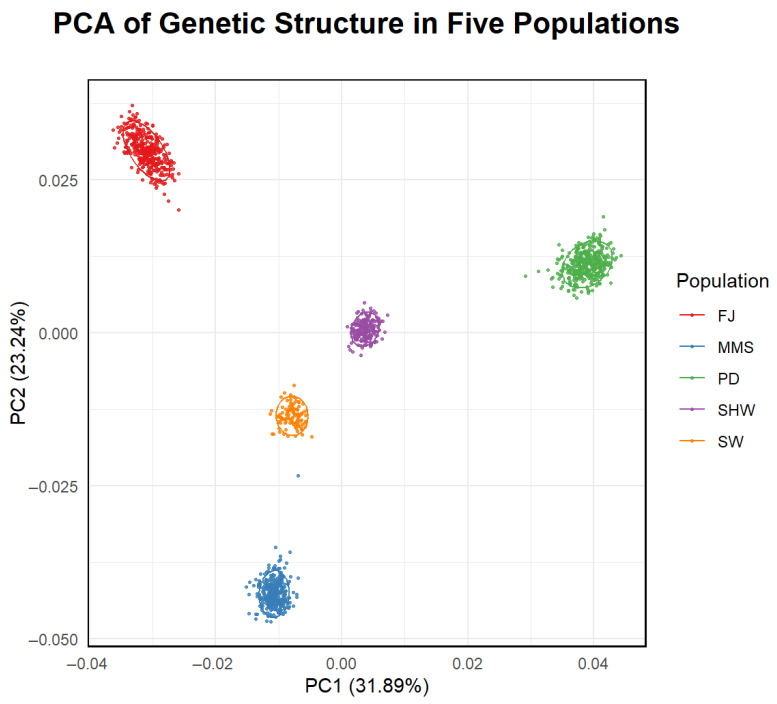
PCA of five pig populations based on 60K SNPs genotype data.

Principal component analysis (PCA) revealed clear genetic differentiation among the five local pig breeds from Shanghai, with individuals from each breed forming distinct, non-overlapping clusters. The first two principal components accounted for 31.89% and 23.24% of the total genetic variation, respectively (Figure 5).

### 3.5. ROH Analysis

Analysis of runs of homozygosity (ROH) using PLINK v 1.90 revealed distinct patterns across the five populations (Table 4). The total number of ROHs detected was highest in the PD population (24,269) and lowest in the SW population (3249). The mean length of a single ROH was longest in the PD (10.29 Mb) and FJ (10.27 Mb) populations.

**Figure 6:**
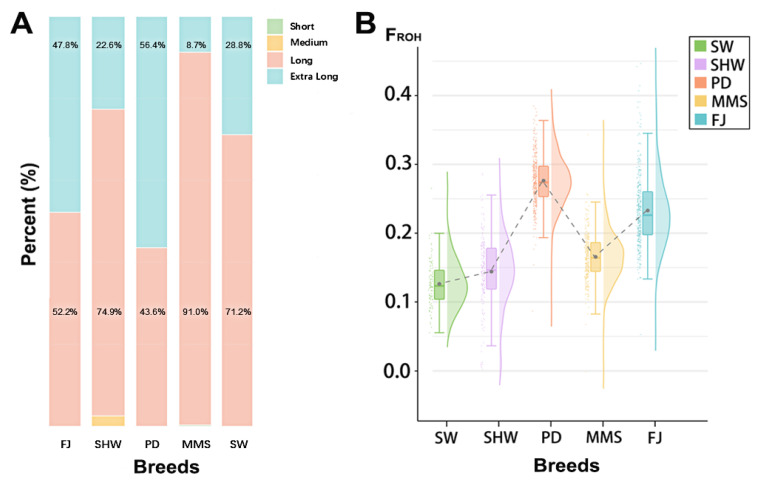
Comparison of genetic inbreeding levels of five local pig breeds populations. (**A**) The length distribution of ROH in every breed; (**B**) comparison of the F_ROH_ among the five pig breeds. The points in the figure represent each individual, and the dotted line represents the connection of the mean values of F_ROH_.

At the individual level, the average number of ROHs per animal ranged from 31.2 (SW) to 61.1 (PD). The mean total ROH length per individual was also highest in the PD population (627.51 Mb) and lowest in the SW population (287.70 Mb), indicating significant inter-population variation. In all breeds, a substantial proportion of ROHs fell into the long (5–10 Mb) and extra-long (>10 Mb) segments (Figure 6).

The genomic inbreeding coefficient (F_ROH_) was derived from individual ROH data. The PD population showed the highest mean F_ROH_ (0.277), followed by FJ (0.234), MMS (0.166), SHW (0.145), and SW (0.127). Notably, several individuals within the FJ and PD populations exhibited particularly high F_ROH_ values (Figure 6).

### 3.6. Comparison of Genetic Diversity in Selected Offspring

For the purebred selection offspring population (MMS_New), the following genetic diversity indices were calculated: Ho = 0.3199, He = 0.3129, PIC = 0.2529, Pi = 0.3132, and MAF = 0.2302. Compared to the original conservation population (Table 4), the offspring population showed higher levels of genetic diversity across these metrics. These results demonstrate that pedigree analysis facilitated by genotyping chips, combined with informed purebred selection, can effectively enhance genetic diversity, reduce inbreeding, and improve the overall effectiveness of conservation breeding programs.

## 4. Discussion

This study presents the first application of the independently developed “Shenxin I” 60K single-nucleotide polymorphism (SNP) liquid-phase chip for Shanghai local pig populations. Utilizing this novel tool, a systematic analysis was conducted to assess the genetic diversity, population structure, inbreeding levels, and genomic breed composition of five representative local pig breeds (FJ, MMS, PD, SHW, and SW) and an offspring population (MMS_NEW). The findings not only elucidate the current genetic status of Shanghai’s indigenous pig breeds but also establish a critical theoretical foundation for their subsequent conservation and selective breeding programs. The genotyping by target sequencing (GBTS)-based liquid-phase chip demonstrates significant advantages for SNP genotyping in both plant and animal studies, particularly in terms of accuracy, reproducibility, and cost-effectiveness. For instance, the GenoBaits WheatSNP16K chip exhibited high reproducibility, with genotypic consistency between two batches ranging from 99.29% to 99.62% across ten cultivars [28]. Similarly, Zhang et al. developed a porcine GBTS50K chip that achieved high SNP call rates (0.997–0.998) and demonstrated higher accuracies than the popular GGP-Porcine bead chip for genomic selection of two important traits [13]. In the initial development phase of the ‘Shenxin I’ chip, the study validated its performance across five local pig breeds in Shanghai. The chip achieved a high SNP call rate of 99.6%, confirming its suitability and reliability for genetic diversity evaluation in these local pig populations.

Genomic variation within populations provides the fundamental substrate for species evolution, manifesting phenotypically as population genetic diversity [29]. Investigations into the genetic architecture of indigenous pig breeds are crucial for elucidating their phylogenetic origins, evolutionary trajectories, and inter-breed genetic affinities. These insights form the scientific cornerstone for developing effective conservation strategies and sustainable utilization programs for these invaluable porcine genetic resources [30]. Traditionally, the evaluation of genetic diversity in livestock has relied heavily on microsatellite markers. However, with recent technological advancements, SNP chips have demonstrated significant advantages. Research indicates that estimating kinship with SNP markers achieves higher accuracy than with microsatellite markers. Furthermore, SNPs offer greater coverage density, broader genomic distribution, higher abundance, lower mutation rates, and superior genetic stability [31,32].

In this study, the results of PCA revealed distinct breed clustering patterns among the five local pig breeds in Shanghai, indicating significant genetic differences between populations. The expected heterozygosity (He) of five local pig breeds ranged from 0.2966 to 0.3295, while observed heterozygosity (Ho) ranged from 0.2954 to 0.3385. Compared with other local pig breeds in China, these values were lower than those of Licha Black Pig (Ho = 0.3576) [4], Dingan Pig (Ho = 0.34) [33], and Jiangshan Black Pig (Ho = 0.345) [34], but higher than Tongcheng Pig (Ho = 0.2185) and Rongchang Pig (Ho = 0.1978) [35], placing them at an intermediate level. The nucleotide diversity (Pi) of the five groups ranged from 0.3051 to 0.3326. This was significantly higher than that of several Western commercial pig breeds, such as Duroc (Pi = 0.2042) and Landrace (Pi = 0.2298) [36]. It indicates that the local pig breeds in Shanghai still retain a high level of genetic diversity at the genomic level.

Runs of homozygosity (ROH) represent extended homozygous segments within the genome. ROH of varying lengths correlate with an individual’s kinship background relative to a common ancestor. The length and number of ROH within a population can reflect its demographic history and genomic breed composition. Excessively long ROH can reduce population genetic diversity and serve as an indicator for assessing inbreeding levels in livestock [37,38]. In this study, the average inbreeding coefficients (F_ROH_) calculated from ROH for the FJ, MMS, PD, SHW, and SW groups were 0.234, 0.166, 0.277, 0.145, and 0.127, respectively. Individual inbreeding coefficients were notably higher in the FJ and PD populations compared to the other groups. These elevated F_ROH_ values suggest a critical need to introduce new genetic material, expand the effective population size, and strategically manage mating to avoid inbreeding in future conservation programs. In the linkage disequilibrium (LD) decay analysis, the PD population exhibited higher mean LD at the same SNP distance and a slower decay rate compared to other groups. The rate of LD decay is influenced by selection intensity, breeding strategies, and genetic background. Typically, populations subjected to stronger domestication and selective pressures demonstrate slower LD decay. Similarly, the decline in population genetic diversity in indigenous pig breeds due to natural selection and genetic drift also slows the rate of LD decay [39,40].

The genomic breed composition (GBC) analysis revealed that certain individuals within the FJ, PD, MMS, and SHW groups exhibited a genomic purity of less than 95%, while the SW group demonstrated overall high genetic purity. This finding underscores the critical need for implementing routine purity screening within these conservation populations. Overall, the GBC results indicate that the conservation programs for these breeds have been largely successful in maintaining genomic integrity. However, the detected introgression in some individuals suggests that unplanned crossbreeding or errors in pedigree recording may have occurred. To ensure the long-term genetic integrity of these valuable populations, it is crucial to strengthen pedigree file management and implement ongoing gene-assisted monitoring protocols [41].

Analysis of the Meishan pig breeding population (MMS_NEW) revealed that it exhibits higher observed heterozygosity (Ho = 0.3199) and expected heterozygosity (He = 0.3129) than the original conservation population (MMS). Concurrently, the average inbreeding coefficient (FROH = 0.154) was slightly lower than that of the MMS group (F_ROH_ = 0.166). The results demonstrate that the current breeding strategy has successfully enhanced production performance without compromising genetic diversity. In fact, the program has facilitated a moderate expansion of the genetic base. When combined with the genomic breed composition (GBC) identification results, these findings confirm that the selected breeding population has successfully introduced beneficial genetic variations or optimized allelic combinations while rigorously maintaining breed purity.

In summary, this study employed high-throughput liquid-phase chip technology to conduct a comprehensive assessment of the genetic diversity within Shanghai’s indigenous pig populations. The analysis identified the diverse genetic bottlenecks and inbreeding risks faced by different breeds and validated the positive genetic outcomes of the ongoing Meishan pig breeding program. This study is not without limitations. Although the total sample size was substantial (N = 1451), the unequal sample sizes across populations may have affected the statistical precision of some comparative analyses. Despite this, the findings provide crucial scientific evidence for formulating differentiated conservation strategies, optimizing breeding programs, and safeguarding the security of these critical genetic resources. This research holds significant demonstrative value for promoting the sustainable utilization and conservation of China’s local livestock genetic resources.

## 5. Conclusions

This study employed the ‘Shenxin I’ 60K liquid-phase chip to analyze the genetic diversity and population structure of five groups: FJ, MMS, PD, SHW, and SW. The results demonstrate that native pig breeds in Shanghai retain considerable genetic diversity and exhibit clear distinctions among populations. genomic breed composition (GBC) analysis proved to be an effective tool for monitoring genomic introgression within core conservation populations, offering a robust technical approach for purebred breeding initiatives. However, elevated inbreeding coefficients were observed in certain individuals from the PD and FJ populations. This finding underscores the need for implementing effective management strategies, such as expanding the effective population size, to mitigate the risk of genetic diversity loss due to inbreeding. Overall, this research provides crucial theoretical support and practical guidance for the conservation and utilization of Shanghai’s indigenous pig genetic resources.

## Figures and Tables

**Table 1:** Sample size and sources.

Breed	Numbers	Source of Samples
FJ	364	Shanghai Qinnong Animal Husbandry Technology Co., Ltd. and Shanghai Songlin Food (Group) Co., Ltd. (Shanghai, China)
MMS	333	Shanghai Jiading Meishan Pig Breeding Centre (Shanghai, China)
PD	397	Shanghai Puhui Superior Seed Breeding Technology Co., Ltd. (Shanghai, China)
SHW	199	Shanghai Academy of Agricultural Sciences Zhuanghang Comprehensive Experimental Station (Shanghai, China
SW	104	Shanghai Shawutou Agricultural Technology Co., Ltd. (Shanghai, China)
MMS_NEW	54	Shanghai Jiading Meishan Pig Breeding Centre

**Table 2:** Information on 1248 samples from twenty-five pig breeds.

Area	Breed	Code	Sample Size
Foreign	Duroc pig Landrace pig Yorkshire pig Pietrain pig Berkshire pig	D L Y P B	78 35 72 20 16
Shanghai	Zhogmeishan pig Fengjing pig Shawutou pig Pudongbai pig Shanghaibai pig	MMS FJ SW PD SHW	124 32 95 89 25
Jiangsu province	Dongchuan pig Erhualian pig Huaibei pig Hongdenglong pig Shan pig Jiangquhai pig Xiaomeishan pig Mi pig	DC EH HB HD SZ JQ SMS MI	10 42 34 30 10 38 75 36
Zhejiang province	Bihu pig Chuanhua pig Chaluhei pig Jiaxinghei pig Lanxihua pig Jinhuaertouwu pig Shengxianhua pig	BH CA CL JX LX JHL SH	29 59 22 88 40 57 62
Total			1248

**Table 3:** Genetic diversity parameters of five pig breeds from Shanghai, China.

Breed	MAF	Ho	He	Pi	Pn	PIC
FJ	0.2233 ± 0.1455	0.2954 ± 0.1559	0.2966 ± 0.1543	0.3051 ± 0.1468	0.5202	0.2478 ± 0.1035
MMS	0.2304 ± 0.1455	0.3123 ± 0.1587	0.3077 ± 0.1538	0.3124 ± 0.1548	0.5308	0.2516 ± 0.1118
PD	0.2432 ± 0.1334	0.3385 ± 0.1529	0.3295 ± 0.1460	0.3326 ± 0.1344	0.4876	0.2682 ± 0.0948
SHW	0.2438 ± 0.1479	0.3309 ± 0.1608	0.3241 ± 0.1540	0.3250 ± 0.1543	0.7844	0.2603 ± 0.1111
SW	0.2301 ± 0.1402	0.3248 ± 0.1611	0.3137 ± 0.1507	0.3148 ± 0.1444	0.5976	0.2550 ± 0.1022

Note: FJ, Fengjing pigs; MMS, Meishan pigs; PD, Pudong White pigs; SHW, Shanghai White pigs; SW, Shawutou pigs. MAF, minor allele frequency; He, expected heterozygosity; Ho, observed heterozygosity; Pn, polymorphic marker ratio; Pi, nucleotide diversity. PIC, Polymorphism Information Content.

**Table 4:** MMS_NEW population genetic diversity index.

Parameters	MMS	MMS_NEW	Mann–Whitney Test
MAF	0.2304 ± 0.1455	0.2302 ± 0.1437	<0.05
Ho	0.3123 ± 0.1587	0.3199 ± 0.1649	<0.0001
He	0.3077 ± 0.1538	0.3129 ± 0.1496	<0.05
Pi	0.3124 ± 0.1548	0.3132 ± 0.1502	<0.05
PIC	0.2516 ± 0.1118	0.2529 ± 0.1078	<0.05

## Data Availability

The original contributions presented in this study are included in the article. Further inquiries can be directed to the corresponding authors.
